# Author Correction: Global influence of soil texture on ecosystem water limitation

**DOI:** 10.1038/s41586-025-08975-3

**Published:** 2025-04-23

**Authors:** Fabian J. P. Wankmüller, Louis Delval, Peter Lehmann, Martin J. Baur, Andrea Cecere, Sebastian Wolf, Dani Or, Mathieu Javaux, Andrea Carminati

**Affiliations:** 1 Institute of Terrestrial Ecosystems, ETH Zurich, Zurich, Switzerland; 2 Earth and Life Institute, Environmental Sciences, UCLouvain, Ottignies-Louvain-la-Neuve, Belgium; 3 Department of Geography, University of Cambridge, Cambridge, UK; 4 Conservation Research Institute, University of Cambridge, Cambridge, UK; 5 Department of Civil and Environmental Engineering, University of Nevada, Reno, NV USA; 6 Agrosphere IBG-3, Forschungszentrum Jülich, Jülich, Germany

**Keywords:** Hydrology, Hydrology, Climate and Earth system modelling, Ecophysiology, Ecophysiology

Correction to: *Nature* 10.1038/s41586-024-08089-2 Published online 23 October 2024

In the version of this article initially published, incorrect values were shown in the “h_b_/[cm]” column of Supplementary Table 1, and the last sentence of the legend for Extended Data Fig. 3 had an error where “pale colours” now replaces “dotted area, AI > 1” in the description. The colours for loamy sand and clay in Fig. 1 have been updated to match usage in other figures, while the full names of the authors have now been spelled out. The changes have been made in the Supplementary information and HTML and PDF versions of the article.

