# Supplementary material for: Global influence of soil texture on ecosystem water limitation
Source: Nature. 2024 Oct 23;635(8039):631–8. doi: 10.1038/s41586-024-08089-2 (PMC11578876; doi:10.1038/s41586-024-08089-2)
Supplement: Supplementary file 1 — Supplementary Figs. 1–11 and Tables 1 and 2. [file 41586_2024_8089_MOESM1_ESM.pdf]

nature portfolio

https://doi.org/10.1038/s41586-024-08089-2

Supplementary information

Global influence of soil texture on ecosystem water limitation

In the format provided by the authors and unedited

SUPPLEMENTARY INFORMATION

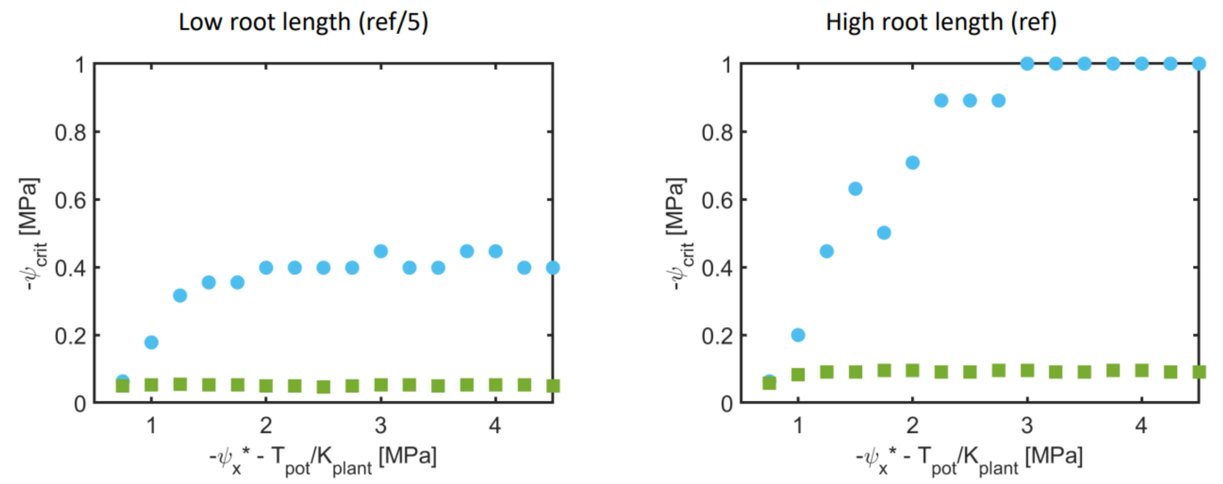

Fig. S1 | Sensitivtiy of ψ_{crit} on plant traits across two contrasting soil textures (clay in blue and loamy sand in green) for two root lengths (the reference one used for the simulations of Fig2 and one with 5 times less roots). ψ_x* is the plant water potential threshold at which plant conductance drops (e.g. due to cavitation). A key variable to explain the sensitivity of ψ_{crit} to soil texture is: $-T_{pot}/K_{plant} - \psi_{x*}$. When it is low (< ca. 1 MPa), plants limit transpiration and there are no effects of soil texture on ψ_{crit}. The effects of soil texture are visible when $-T_{pot}/K_{plant} - \psi_{x*} > 1$ MPa.

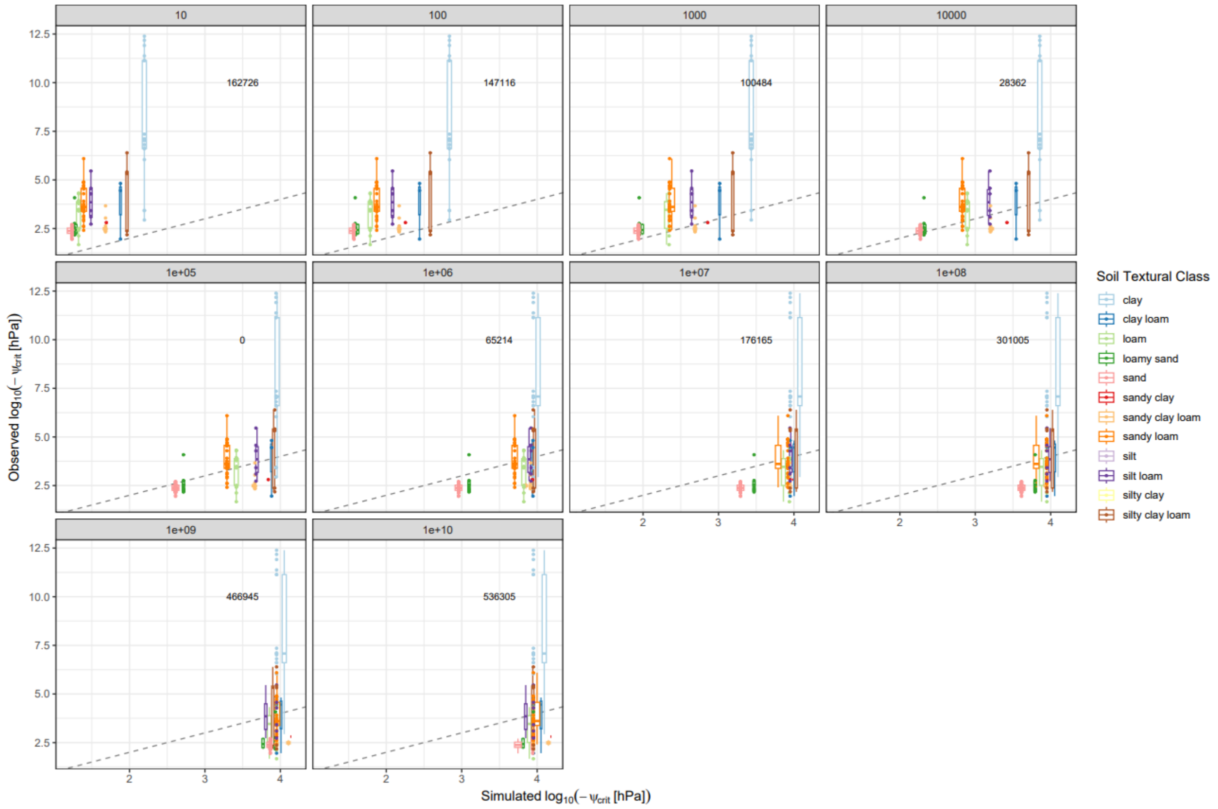

Fig. S2 | Sensitivity of of ψ_{crit} on the effective root length L_{root}. Changing L_{root} from 10 to 1e+10 cm m^{-2} (titles to each panel) results in variable soil texture dependence of ψ_{crit}, i.e. it shows how extraordinary root lengths (~ L_{root} inf.) result in simulated ψ_{crit} being independent from soil texture and converging towards the plant water potential threshold (ψ_{x*}), see also Fig.1d and Fig. S3.

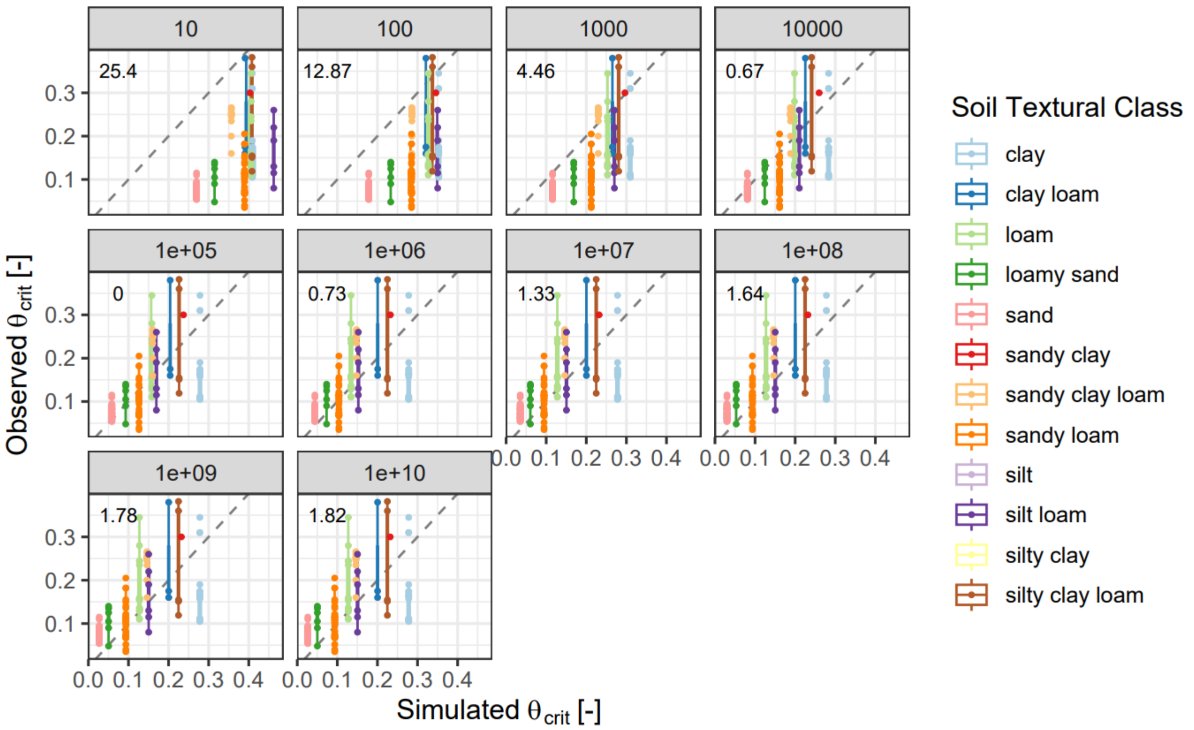

Fig. S3 | Sensitivity of θ_{crit} on the effective root length L_{root}. Changing L_{root} from 10 to 1e+10 cm m^{-2} (titles to each panel) shows how well simulated θ_{crit} fit to observed θ_{crit} (particularly bad for short roots). See also Fig. S2 for comparison.

Table S1 | Hydraulic properties of the soil textural classes used for the calculations of the critical water content θ_{crit}. Saturated θ_{sat} and residual water content θ_{res}, shape parameter l, and air-entry value h_b according to the Brooks and Corey model were chosen from [77]. The power-law exponent of the conductivity function τ was set to τ = 3l+2. For the saturated hydraulic conductivity K_{sat}, the values of [77], K_{sat}^a, and [78], K_{sat}^b, are listed. For the simulations of θ_{crit}, data from [78] were used due to the larger data set and given that variation of K_{sat} were provided (missing in [77]). Note that [77] did not list silt soil textural class and we chose the same values as for silt loam (as it is proposed in Hydrus software).

Soil Textural Class	θ_{sat} [cm^3 cm^{-3}]	θ_{res} [cm^3 cm^{-3}]	l [-]	τ [-]	h_b [cm]	K_{sat}^a [cm h^{-1}]	K_{sat}^b [cm h^{-1}]	No. of fluxnet sites per class	No. of sapfluxnet sites per class
sand	0.437	0.020	0.592	3.776	7.26	21.00	20.08	5	4
loamy sand	0.437	0.035	0.474	3.422	8.69	6.11	4.18	6	0
sandy loam	0.453	0.041	0.322	2.966	14.66	2.59	2.13	9	4
loam	0.463	0.027	0.220	2.660	11.15	1.32	1.58	3	2
silt loam	0.501	0.015	0.211	2.633	20.76	0.68	0.97	6	0
sandy clay loam	0.398	0.068	0.250	2.750	28.08	0.43	0.76	3	1
clay loam	0.464	0.075	0.194	2.582	25.89	0.23	2.06	3	0
silty clay loam	0.471	0.040	0.151	2.453	32.56	0.15	1.36	1	1
sandy clay	0.430	0.109	0.168	2.504	29.17	0.12	0.59	1	0

silty clay	0.479	0.056	0.127	2.381	34.19	0.09	4.42	0	0
clay	0.475	0.090	0.131	2.393	37.3	0.06	2.03	6	2
silt	0.501	0.015	0.211	2.633	20.76	0.68	0.55	0	0

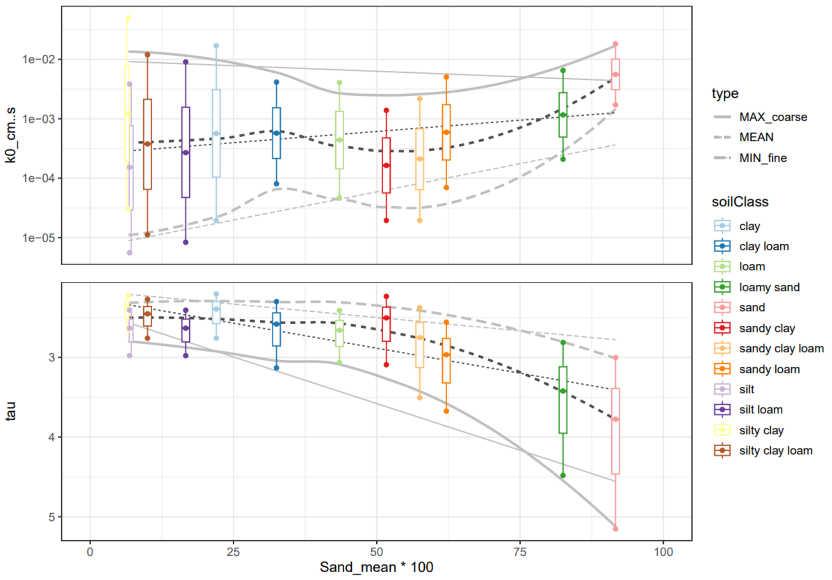

Fig. S4 | Visualization of the variability of two key soil hydraulic properties. Variability of saturated hydraulic conductivity (k0_cm..s, cm s^{-1}) and the slope of the unsaturated hydraulic conductivity curve (tau) within a soil textural class plotted against the mean sand fraction (Sand_mean * 100, %). See the methods section for description how the variability of soil texture class-specific hydraulic properties was derived.

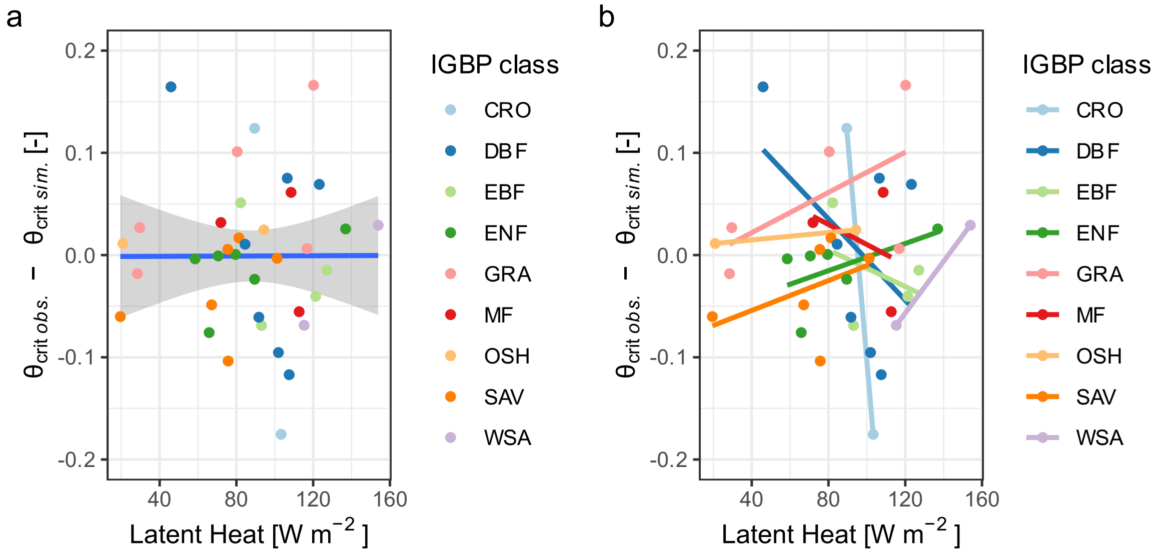

Fig. S5 | Simulated fluxnet θ_{crit} do not show systematic deviation from observed θ_{crit} across climates and biomes. Relationships of the differences between observed (fluxnet) and simulated θ_{crit} to site-specific latent heat fluxes (i.e., to the absolute evapotranspiration rates determined by the climate of each site; T_{pot}) across climates and biomes using the average latent heat flux in the EF-plateau above θ_{crit}.

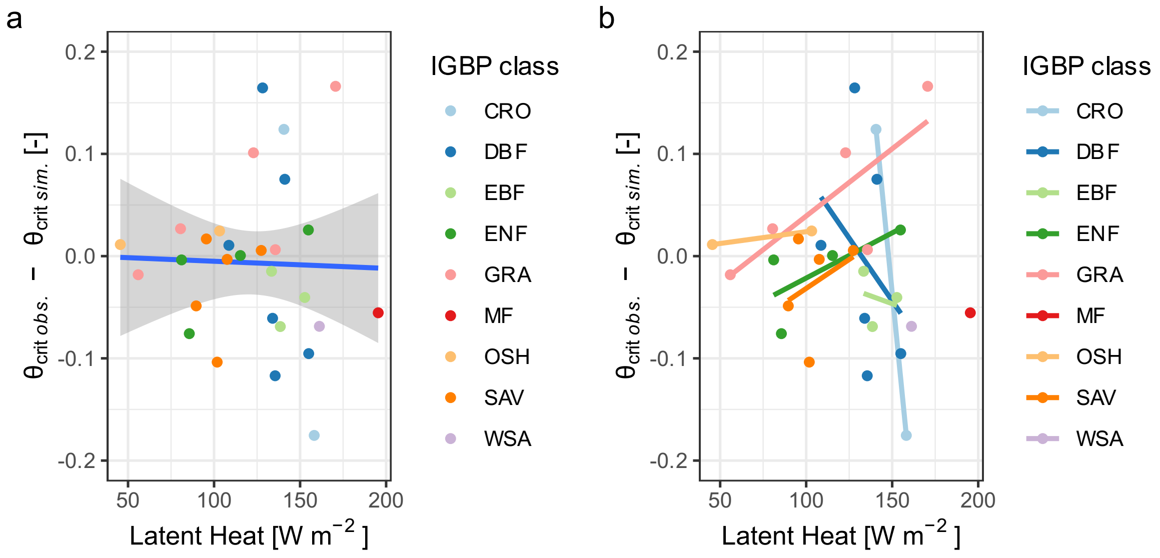

Fig. S6 | Simulated fluxnet θ_{crit} do not show systematic deviation from observed θ_{crit} across climates and biomes. Relationships of the differences between observed (fluxnet) and simulated θ_{crit} to site-specific latent heat fluxes (i.e., to the absolute evapotranspiration rates determined by the climate of each site; T_{pot}) across climates and biomes using the 'envelope' of latent heat fluxes in the EF-plateau above θ_{crit}.

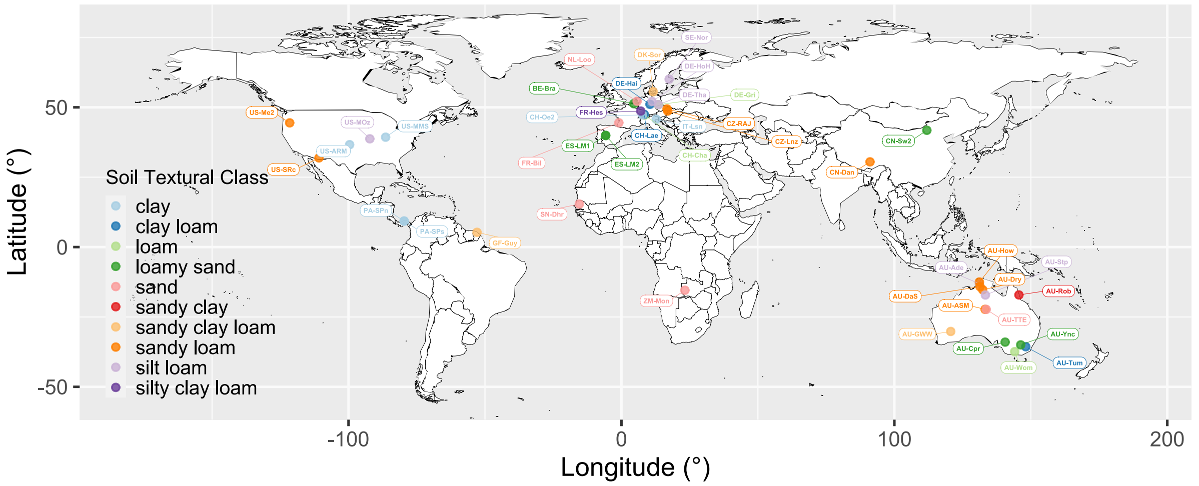

Fig. S7 | Locations of the FLUXNET Eddy-Covariance sites included in this study.

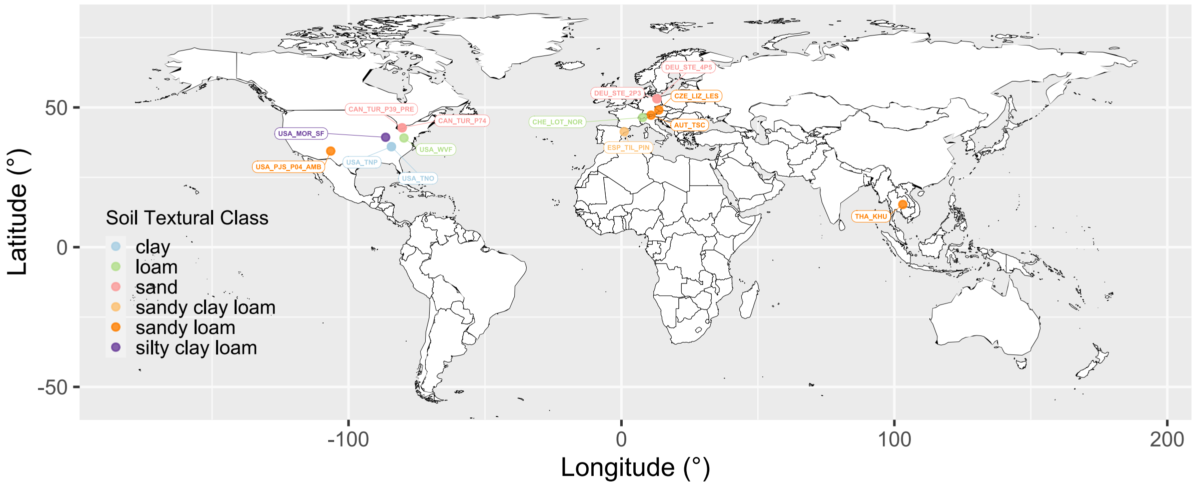

Fig. S8 | Locations of the SAPFLUXNET sites included in this study.

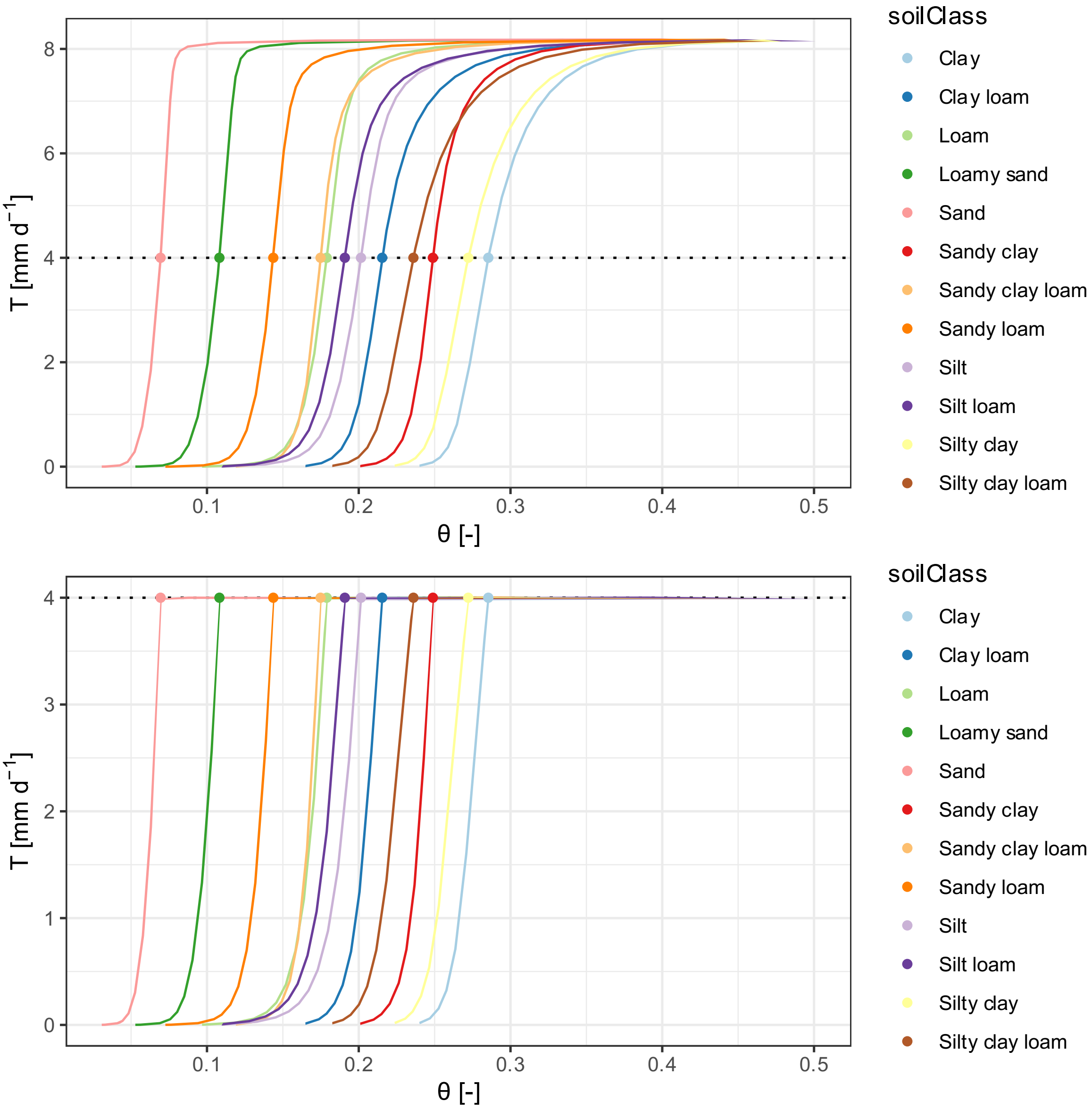

Fig. S9 | Numerical T(θ) functions used for the analysis on the global sensitivity of critical soil moisture thresholds to climate change. Note that the model parameters used in this simulation were slightly different from the default parameter values used in the main analysis of the manuscript (Table S1), but differences in $\Delta\theta_{crit}/\Delta T_{pot}$ around θ_{crit} between this simulation run and the default simulation are negligible in relation to the uncertainties originating from this simplified analysis of climate change impacts (i.e. considering solely the effect of VPD on critical soil water thresholds). Here, $\psi_{leaf\text{-}max}$ = -0.5 MPa, ψ_{x*} = -2 MPa, and L_{root} = 320 m m^{-2} as it was fitted to FLUXNET data only (true ecosystem scale flux data). Note that these parameters ensured that the T(θ) functions did not yet reach their hydraulically restricted plateau in the range of VPD increases projected by the considered climate scenario for 2060-2069 (T_{pot} +65% = 6.6 mm d^{-1}), whereas the default parameter values (Table S1) would hydraulically limit the transpiration rate beforehand.

Table S2 | Site identifier (Site ID), data type (EC: Eddy Covariance, SF: Sapflow), continent, latitude (Lat, °), longitude (Long, °), IGBP land cover class (IGBP), mean annual temperature (MAT, °C) or temperature range (TR, °C) when MAT is not available, mean annual precipitation (MAP, mm), average site-specific latent heat flux where $\theta > \theta_{crit}$, fluxnet only (LE_avg, W m^{-2}), study periods (Periods), locally measured fractions of sand silt and clay (Sand|Silt|Clay, %) where available, soil textural class (soil texture) classified according to USDA where applicable, estimated soil moisture threshold (θ_{crit}, %), median per sapfluxnet site, and references (Ref) of sites used in the study. Abbreviations: WSA, Woody Savannah; SAV, Savannah; EBF, Evergreen Broadleaf Forests; GRA, Grasslands; MF, Mixed Forests; CRO, Croplands; ENF, Evergreen Needleleaf Forests; DBF, Deciduous Broadleaf Forests; OSH, Open Shrublands.

ID	Site ID	Data Type	Continent	Lat	Long	IGBP	MAT *(TR)*	MAP	LE_avg	Periods	Sand|Silt|Clay	Soil texture	θ_{crit}	Ref
1	AU-Ade	EC	Oceania	-13.08	131.12	WSA	*(16 – 36)*	1730	153.9	2007-2009		Silt loam	22	88
2	AU-ASM	EC	Oceania	-22.28	133.25	SAV	*(-4 – 46)*	305.09	67.1	2010-2014	74|11|15	Sandy loam	9.5	89
3	AU-Cpr	EC	Oceania	-34.00	140.59	SAV	*(12 – 45)*	240	19.5	2010-2014		Loamy sand	4.8	90
4	AU-DaS	EC	Oceania	-14.16	131.39	SAV	27.22	975.82		2008-2014		Sandy loam	3.5	91
5	AU-Dry	EC	Oceania	-15.26	132.37	SAV	*(14 – 37)*	895	75.7	2008-2014		Sandy loam	4	92
6	AU-GWW	EC	Oceania	-30.19	120.65	SAV	*(5 – 33)*	240		2013-2014	57|15|28	Sandy clay loam	20	93
7	AU-How	EC	Oceania	-12.49	131.15	WSA	27.01	1449.35	115.3	2001-2014		Sandy loam	7.5	94
8	AU-Rob	EC	Oceania	-17.12	145.63	EBF	*(3 – 33)*	2236	82.2	2014-2014	46|18|36	Sandy clay	30	95
9	AU-Stp	EC	Oceania	-17.15	133.35	GRA	*(11 – 39)*	640		2008-2014		Silt loam	8	96
10	AU-TTE	EC	Oceania	-22.29	133.64	GRA	*(-4 – 46)*	305		2012-2014	91|8|1	Sand	11	97
11	AU-Tum	EC	Oceania	-35.66	148.15	EBF	10.72	1159.01	121.3	2001-2014		Clay loam	17.5	98
12	AU-Wom	EC	Oceania	-37.42	144.09	EBF	*(1 – 30)*	650	93.1	2010-2014	45|29|26	Loam	11	99
13	AU-Ync	EC	Oceania	-34.99	146.29	GRA	*(12 – 37)*	465	28.4	2012-2014		Loamy sand	9	100
14	BE-Bra	EC	Europe	51.31	4.52	MF	9.8	750	71.9	1996-2018		Loamy sand	14	101

**15**	**CH-Cha**	**EC**	**Europe**	**47.21**	**8.41**	**GRA**	**9.5**	**1136**	**120.2**	**2005-2018**		**Loam**	**34.5**	102
**16**	**CH-Lae**	**EC**	**Europe**	**47.48**	**8.36**	**MF**	**8.3**	**1100**	**112.7**	**2004-2018**		**Clay loam**	**16**	103
**17**	**CH-Oe2**	**EC**	**Europe**	**47.29**	**7.73**	**CRO**	**9.8**	**1155**	**103.3**	**2004-2018**	**25|33|42**	**Clay**	**11**	104
**18**	**CN-Dan**	**EC**	**Asia**	**30.50**	**91.07**	**GRA**	**-1.54**	**246.88**	**116.8**	**2004-2005**	**67|18|15**	**Sandy loam**	**15**	105
**19**	**CN-Sw2**	**EC**	**Asia**	**41.79**	**111.90**	**GRA**	**3.4**	**180**	**29.6**	**2010-2012**		**Loamy sand**	**13.5**	106
**20**	**CZ-Lnz**	**EC**	**Europe**	**48.68**	**16.95**	**MF**	**9.80**	**518.03**	**108.5**	**2015-2018**		**Sandy loam**	**20.5**	107
**21**	**CZ-RAJ**	**EC**	**Europe**	**49.44**	**16.70**	**ENF**	**7.1**	**681**	**58.5**	**2012-2018**		**Sandy loam**	**14**	108
**22**	**DE-Gri**	**EC**	**Europe**	**50.95**	**13.51**	**GRA**	**7.8**	**901**	**80.4**	**2004-2018**		**Loam**	**28**	109
**23**	**DE-Hai**	**EC**	**Europe**	**51.08**	**10.45**	**DBF**	**8.3**	**720**	**45.9**	**2000-2018**		**Clay loam**	**38**	110
**24**	**DE-HoH**	**EC**	**Europe**	**52.09**	**11.22**	**DBF**	**9.1**	**563**	**91.7**	**2015-2018**		**Silt loam**	**13**	111
**25**	**DE-Tha**	**EC**	**Europe**	**50.96**	**13.57**	**ENF**	**8.2**	**843**	**70.5**	**1996-2018**		**Silt loam**	**19**	112
**26**	**DK-Sor**	**EC**	**Europe**	**55.59**	**11.64**	**DBF**	**8.2**	**660**	**106.5**	**1996-2018**		**Sandy clay loam**	**25**	113
**27**	**ES-LM1**	**EC**	**Europe**	**39.94**	**-5.78**	**SAV**	**16**	**700**	**101.2**	**2014-2018**		**Loamy sand**	**10.5**	114
**28**	**ES-LM2**	**EC**	**Europe**	**39.93**	**-5.78**	**SAV**	**16**	**700**	**81.3**	**2014-2018**		**Loamy sand**	**12.5**	114
**29**	**FR-Bil**	**EC**	**Europe**	**44.49**	**-0.96**	**ENF**	**12.8**	**930**	**136.9**	**2014-2018**		**Sand**	**9.5**	115
**30**	**FR-Hes**	**EC**	**Europe**	**48.67**	**7.06**	**DBF**	**9.2**	**820**	**107.5**	**2014-2018**		**Silty clay loam**	**11.9**	116
**31**	**GF-Guy**	**EC**	**South America**	**5.28**	**-52.92**	**EBF**	**25.7**	**3041**	**127.1**	**2004-2014**	**48-64| - |43-26**	**Sandy clay loam**	**16**	117
**32**	**IT-Lsn**	**EC**	**Europe**	**45.75**	**12.75**	**OSH**	**13.1**	**1083**	**94.3**	**2016-2018**		**Clay**	**31**	111

**33**	**NL-Loo**	**EC**	**Europe**	**52.17**	**5.74**	**ENF**	**9.8**	**786**	**79.5**	**1996-2018**		**Sand**	**7**	[118]
**34**	**PA-SPn**	**EC**	**South America**	**9.32**	**-79.63**	**DBF**	**26.5**	**2350**		**2007-2009**	**4|30|65**	**Clay**	**31**	[119]
**35**	**PA-SPs**	**EC**	**South America**	**9.31**	**-79.63**	**DBF**	**26.5**	**2350**		**2007-2009**	**4|30|65**	**Clay**	**34.5**	[119]
**36**	**SE-Nor**	**EC**	**Europe**	**60.09**	**17.48**	**ENF**	**5.5**	**527**	**65.8**	**2014-2018**		**Silt loam**	**11.5**	[111]
**37**	**SN-Dhr**	**EC**	**Europe**	**15.40**	**-15.43**	**SAV**	**29**	**404**	**75.5**	**2010-2013**	**95|4.6|0.4**	**Sand**	**7.5**	[120]
**38**	**US-ARM**	**EC**	**North America**	**36.64**	**-99.60**	**CRO**	**14.76**	**843**	**89.5**	**2003-2020**	**28|29|43**	**clay**	**36**	[121]
**39**	**US-Me2**	**EC**	**North America**	**44.45**	**-121.56**	**ENF**	**6.28**	**523**	**89.5**	**2002-2020**	**67|26|7**	**Sandy loam**	**12**	[122]
**40**	**US-MMS**	**EC**	**North America**	**39.32**	**-86.41**	**DBF**	**10.85**	**1032**	**101.9**	**1999-2020**	**34|3|63**	**Clay**	**19**	[123]
**41**	**US-MOz**	**EC**	**North America**	**38.74**	**-92.20**	**DBF**	**12.11**	**986**	**123.2**	**2004-2019**		**Silt loam**	**26**	[124]
**42**	**US-SRc**	**EC**	**North America**	**31.91**	**-110.84**	**OSH**	**22**	**330**	**20.9**	**2008-2014**		**Sandy loam**	**15.5**	[125]
**43**	**ZM-Mon**	**EC**	**Africa**	**-15.44**	**23.25**	**DBF**	**25**	**945**	**84.5**	**2000-2009**	**97.5|1.9|0.6**	**Sand**	**8**	[126]
**44**	**AUT_TSC**	**SF**	**Europe**	**47.23**	**10.84**	**ENF**	**8.5**	**694**		**2012**	**54|44|2**	**sandy loam**	**6.8**	walter.oberhuber@uibk.ac.at, gerhard.wieser@uibk.ac.at
**45**	**CAN_TUR_P39_PRE**	**SF**	**North America**	**42.71**	**-80.36**	**ENF**	**9**	**1000**		**2008-2016**	**98|1|1**	**sand**	**8.9**	[127]
**46**	**CAN_TUR_P74**	**SF**	**North America**	**42.71**	**-80.35**	**ENF**	**9**	**1003**		**2008-2016**	**98|1|1**	**sand**	**8.1**	[127]
**47**	**CHE_LOT_NOR**	**SF**	**Europe**	**46.39**	**7.76**	**ENF**	**5**	**716**		**2006-2015**	**48|42|10**	**loam**	**13.6**	[128]
**48**	**CZE_LIZ_LES**	**SF**	**Europe**	**49.07**	**13.68**	**ENF**	**6**	**837**		**2007-2009**	**60.75|30.98|8.27**	**sandy loam**	**18.2**	[129]
**49**	**DEU_STE_2P3**	**SF**	**Europe**	**53.1**	**13**	**DBF**	**8.9**	**595**		**2002-2003**	**92.5|5|2.5**	**sand**	**5.8**	[130]
**50**	**DEU_STE_4P5**	**SF**	**Europe**	**53.1**	**13**	**DBF**	**8.9**	**595**		**2004-2005**	**92.5|5|2.5**	**sand**	**6.4**	[130]
**51**	**ESP_TIL_PIN**	**SF**	**Europe**	**41.33**	**1.01**	**ENF**	**10.1**	**674**		**2005-2011**	**60|20|20**	**sandy clay loam**	**25.3**	[131]

**52**	**THA_KHU**	**SF**	**Asia**	**15.27**	**103.08**	**DBF**	**27.2**	**1178**		**2006-2008**	**65|25|10**	**sandy loam**	**11.1**	[132]
**53**	**USA_MOR_SF**	**SF**	**North America**	**39.32**	**-86.41**	**DBF**	**12**	**1159**		**2011-2013**	**10|60|30**	**silty clay loam**	**15.5**	[133]
**54**	**USA_PJS_P04_AMB**	**SF**	**North America**	**34.39**	**-106.53**	**WSA**	**12.7**	**311**		**2006-2015**	**52|42|6**	**sandy loam**	**10.7**	[134]
**55**	**USA_TNO**	**SF**	**North America**	**35.97**	**-84.28**	**DBF**	**14.6**	**1497**		**1998-1999**		**clay**	**10.7**	[135]
**56**	**USA_TNP**	**SF**	**North America**	**35.96**	**-84.29**	**ENF**	**14.6**	**1489**		**1998-1999**		**clay**	**16.4**	[135]
**57**	**USA_WVF**	**SF**	**North America**	**39.06**	**-79.69**	**DBF**	**9.4**	**1408**		**1998-1999**		**loam**	**15.8**	[135,136]

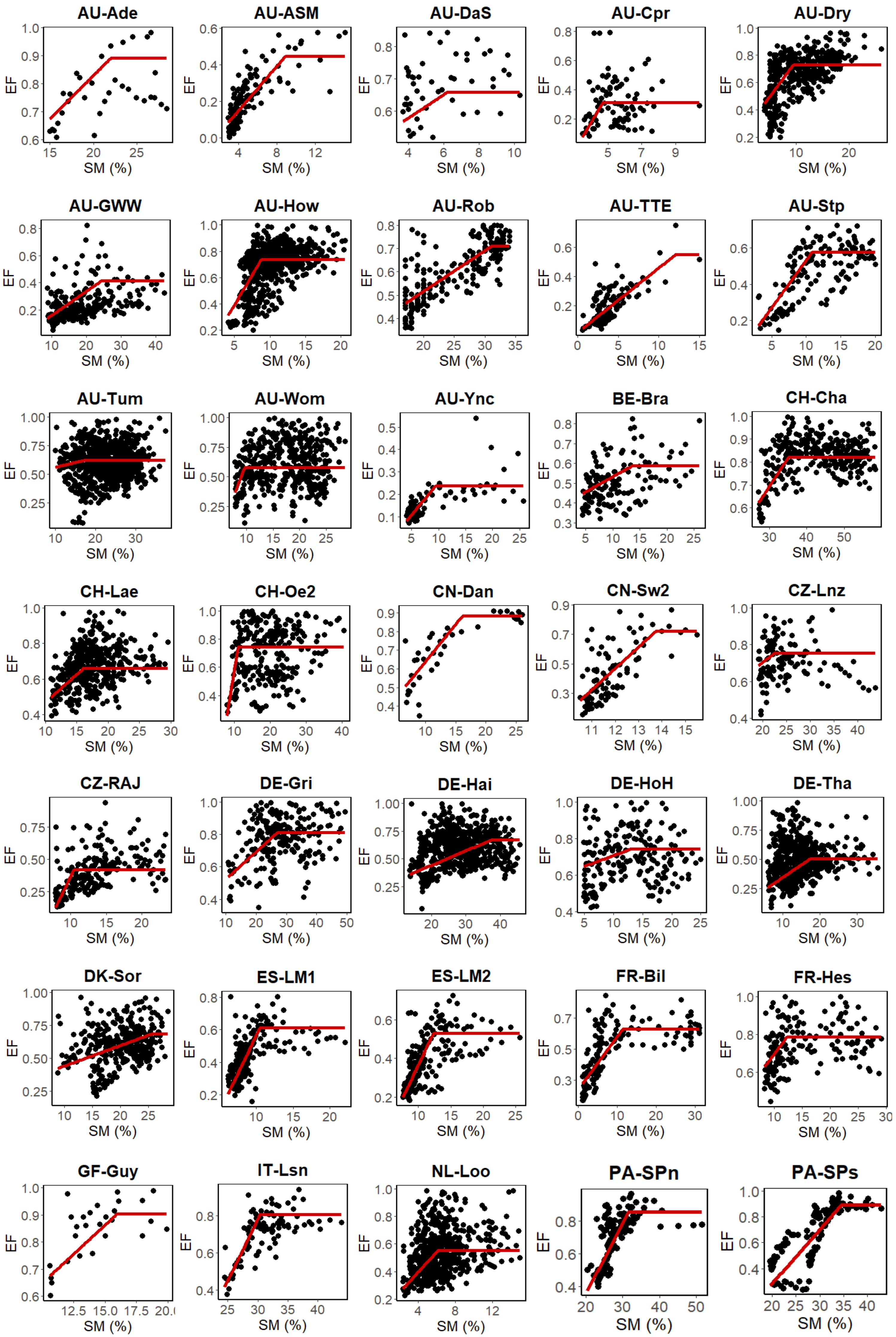
AU-Ade
AU-ASM
AU-DaS
AU-Cpr
AU-Dry
AU-GWW
AU-How
AU-Rob
AU-TTE
AU-Stp
AU-Tum
AU-Wom
AU-Ync
BE-Bra
CH-Cha
CH-Lae
CH-Oe2
CN-Dan
CN-Sw2
CZ-Lnz
CZ-RAJ
DE-Gri
DE-Hai
DE-HoH
DE-Tha
DK-Sor
ES-LM1
ES-LM2
FR-Bil
FR-Hes
GF-Guy
IT-Lsn
NL-Loo
PA-SPn
PA-SPs
EF
SM (%)

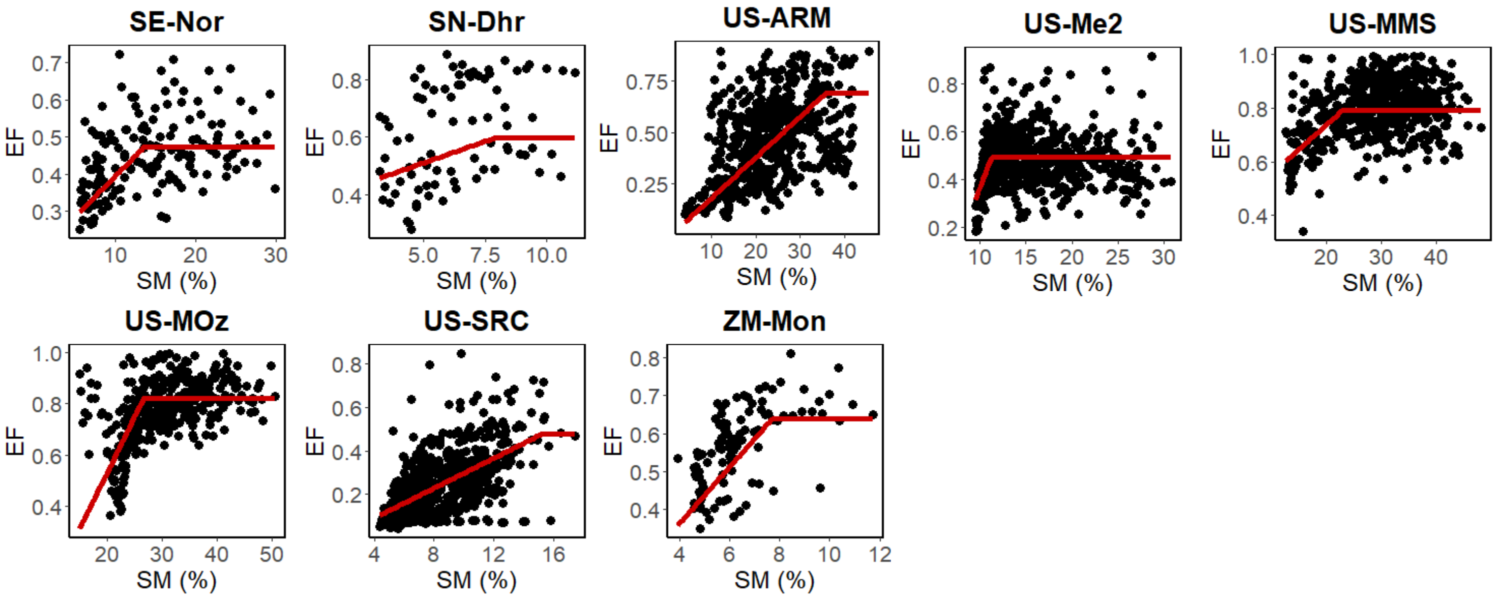

Figure S10 – Fitted EF-SM relationship using a linear-plus-plateau model for the 43 Eddy-Covariance sites used in this study. EF: evaporative fraction (-); SM: soil moisture (%).

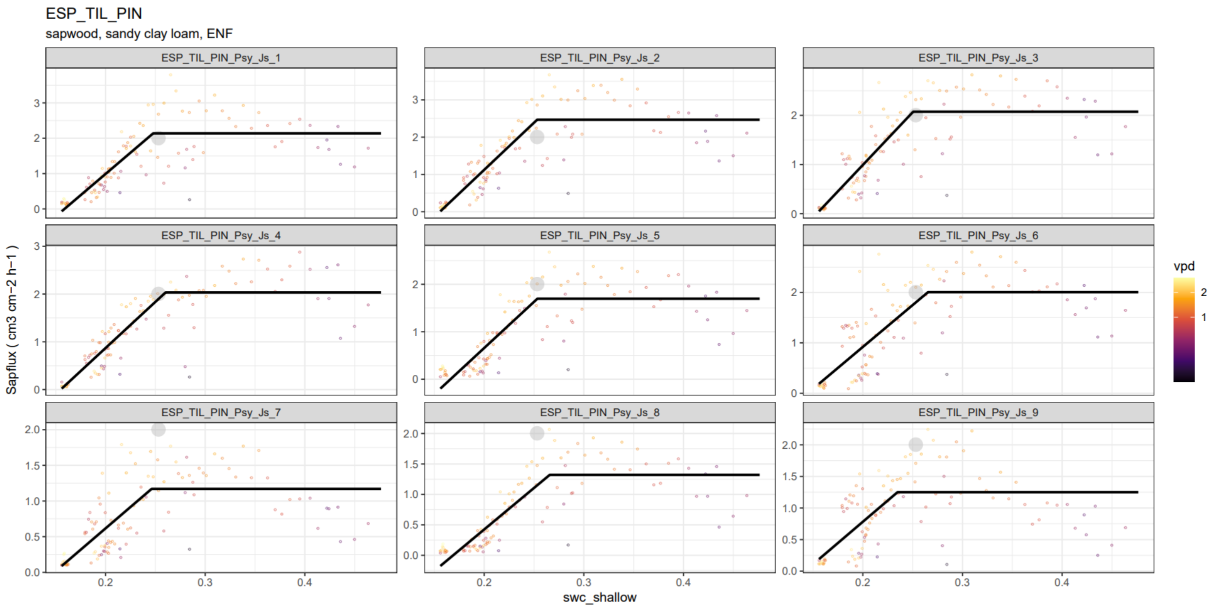

Figure S11 | Fitted Sapflux – soil moisture (swc_shallow, m3/m3) linear-plateau relationship for an exemplary site containing 9 sapflux measurements (trees). Note that in Table S2 the site-specific median soil moisture threshold is reported (indicated by the grey dot located at median θ_{crit} and median sapflux density).

References

88. Beringer, J. *et al.* Special—Savanna Patterns of Energy and Carbon Integrated Across the Landscape. *Bulletin of the American Meteorological Society* **92**, 1467–1485 (2011).

89. Cleverly, J. *et al.* Dynamics of component carbon fluxes in a semi-arid Acacia woodland, central Australia. *Journal of Geophysical Research: Biogeosciences* **118**, 1168–1185 (2013).

90. Meyer, W. S., Kondrlovà, E. & Koerber, G. R. Evaporation of perennial semi-arid woodland in southeastern Australia is adapted for irregular but common dry periods. *Hydrological Processes* **29**, 3714–3726 (2015).

91. Hutley, L. B., Beringer, J., Isaac, P. R., Hacker, J. M. & Cernusak, L. A. A sub-continental scale living laboratory: Spatial patterns of savanna vegetation over a rainfall gradient in northern Australia. *Agricultural and Forest Meteorology* **151**, 1417–1428 (2011).

92. Cernusak, L. A., Hutley, L. B., Beringer, J., Holtum, J. A. M. & Turner, B. L. Photosynthetic physiology of eucalypts along a sub-continental rainfall gradient in northern Australia. *Agricultural and Forest Meteorology* **151**, 1462–1470 (2011).

93. Macfarlane, C., Prober, S. & Wiehl, G. FLUXNET2015 AU-GWW Great Western Woodlands, Western Australia, Australia, Dataset. *FLUXNET* https://fluxnet.org//doi/FLUXNET2015/AU-GWW (2013).

94. Beringer, J., Hutley, L. B., Tapper, N. J. & Cernusak, L. A. Savanna fires and their impact on net ecosystem productivity in North Australia. *Global Change Biology* **13**, 990–1004 (2007).

95. Beringer, J. *et al.* An introduction to the Australian and New Zealand flux tower network – OzFlux. *Biogeosciences* **13**, 5895–5916 (2016).

96. Beringer, J., Hutley, L. B., Hacker, J. M., Neininger, B. & Paw U, K. T. Patterns and processes of carbon, water and energy cycles across northern Australian landscapes: From point to region. *Agricultural and Forest Meteorology* **151**, 1409–1416 (2011).

97. Cleverly, J., Eamus, D. & Isaac, P. FLUXNET2015 AU-TTE Ti Tree East, Dataset. *FLUXNET* https://fluxnet.org//doi/FLUXNET2015/AU-TTE (2012).

98. Leuning, R., Cleugh, H. A., Zegelin, S. J. & Hughes, D. Carbon and water fluxes over a temperate Eucalyptus forest and a tropical wet/dry savanna in Australia: measurements and comparison with MODIS remote sensing estimates. *Agricultural and Forest Meteorology* **129**, 151–173 (2005).

99. Arndt, S. K., Hinko-Najera, N., Griebel, A., Beringer, J. & Livesley, S. FLUXNET2015 AU-Wom Wombat, Dataset. *FLUXNET* https://fluxnet.org//doi/FLUXNET2015/AU-Wom (2010).

100. Yee, M. S. *et al.* A comparison of optical and microwave scintillometers with eddy covariance derived surface heat fluxes. *Agricultural and Forest Meteorology* **213**, 226–239 (2015).

101. Carrara, A., Janssens, I. A., Curiel Yuste, J. & Ceulemans, R. Seasonal changes in photosynthesis, respiration and NEE of a mixed temperate forest. *Agricultural and Forest Meteorology* **126**, 15–31 (2004).

102. Merbold, L. *et al.* Greenhouse gas budget (CO2, CH4 and N2O) of intensively managed grassland following restoration. *Global Change Biology* **20**, 1913–1928 (2014).

103. Etzold, S. *et al.* The Carbon Balance of Two Contrasting Mountain Forest Ecosystems in Switzerland: Similar Annual Trends, but Seasonal Differences. *Ecosystems* **14**, 1289–1309 (2011).

104. Dietiker, D., Buchmann, N. & Eugster, W. Testing the ability of the DNDC model to predict CO2 and water vapour fluxes of a Swiss cropland site. *Agriculture, Ecosystems & Environment* **139**, 396–401 (2010).

105. Shi, P. *et al.* Net ecosystem CO2 exchange and controlling factors in a steppe—Kobresia meadow on the Tibetan Plateau. *SCI CHINA SER D* **49**, 207–218 (2006).

106. Changliang, S. FLUXNET2015 CN-Sw2 Siziwang Grazed (SZWG), Dataset. *FLUXNET* https://fluxnet.org//doi/FLUXNET2015/CN-Sw2 (2010).

107. Gourlez de la Motte, L. *et al.* Non-stomatal processes reduce gross primary productivity in temperate forest ecosystems during severe edaphic drought. *Phil. Trans. R. Soc. B* **375**, 20190527 (2020).

108. McGloin, R. *et al.* Energy balance closure at a variety of ecosystems in Central Europe with contrasting topographies. *Agricultural and Forest Meteorology* **248**, 418–431 (2018).

109. Prescher, A.-K., Grünwald, T. & Bernhofer, C. Land use regulates carbon budgets in eastern Germany: From NEE to NBP. *Agricultural and Forest Meteorology* **150**, 1016–1025 (2010).

110. Knohl, A., Ernst Detlef, S., Kolle, O. & Buchmann, N. Large Carbon Uptake by an Unmanaged 250 year-old Deciduous Forest in Central Germany. *Agricultural and Forest Meteorology* **118**, 151–167 (2003).

111. Drought 2018 Team & ICOS Ecosystem Thematic Centre. Drought-2018 ecosystem eddy covariance flux product in FLUXNET-Archive format - release 2019-1. (2019) doi:10.18160/PZDK-EF78.

112. Grünwald, T. & Bernhofer, C. A decade of carbon, water and energy flux measurements of an old spruce forest at the Anchor Station Tharandt. *Tellus B* **59**, 387–396 (2007).

113. Pilegaard, K., Ibrom, A., Courtney, M., Hummelshøj, P. & Jensen, N. O. Increasing net CO2 uptake by a Danish beech forest during the period from 1996 to 2009. *Agricultural and Forest Meteorology* **151**, 934–946 (2011).

114. El-Madany, T. S. *et al.* Drought and heatwave impacts on semi-arid ecosystems' carbon fluxes along a precipitation gradient. *Philos Trans R Soc Lond B Biol Sci* **375**, 20190519 (2020).

115. Deirmendjian, L. *et al.* Hydro-ecological controls on dissolved carbon dynamics in groundwater and export to streams in a temperate pine forest. *Biogeosciences* **15**, 669–691 (2018).

116. Granier, A. *et al.* The carbon balance of a young Beech forest. *Functional Ecology* **14**, 312–325 (2000).

117. Bonal, D. *et al.* Impact of severe dry season on net ecosystem exchange in the Neotropical rainforest of French Guiana. *Global Change Biology* **14**, 1917–1933 (2008).

118. Gioli, B. *et al.* Comparison between tower and aircraft-based eddy covariance fluxes in five European regions. *Agricultural and Forest Meteorology* **127**, 1–16 (2004).

119. Wolf, S., Eugster, W., Potvin, C., Turner, B. & Buchmann, N. Carbon sequestration potential of tropical pasture compared with afforestation in Panama. *Global Change Biology* **17**, 2763–2780 (2011).

120. Tagesson, T. *et al.* Ecosystem properties of semiarid savanna grassland in West Africa and its relationship with environmental variability. *Glob Chang Biol* **21**, 250–264 (2015).

121. Bagley, J. E. *et al.* The influence of land cover on surface energy partitioning and evaporative fraction regimes in the U.S. Southern Great Plains. *Journal of Geophysical Research: Atmospheres* **122**, 5793–5807 (2017).

122. Campbell, J. L., Sun, O. J. & Law, B. E. Disturbance and net ecosystem production across three climatically distinct forest landscapes. *Global Biogeochemical Cycles* **18**, (2004).

123. Baldocchi, D. D. *et al.* Predicting the onset of net carbon uptake by deciduous forests with soil temperature and climate data: a synthesis of FLUXNET data. *Int J Biometeorol* **49**, 377–387 (2005).

124. Wang, Y. *et al.* Testing stomatal models at the stand level in deciduous angiosperm and evergreen gymnosperm forests using CliMA Land (v0.1). *Geoscientific Model Development* **14**, 6741–6763 (2021).

125. Chu, H. *et al.* Representativeness of Eddy-Covariance flux footprints for areas surrounding AmeriFlux sites. *Agricultural and Forest Meteorology* **301–302**, 108350 (2021).

126. Merbold, L. *et al.* Precipitation as driver of carbon fluxes in 11 African ecosystems. *Biogeosciences* **6**, 1027–1041 (2009).

127. Peichl, M., Arain, M. A. & Brodeur, J. J. Age effects on carbon fluxes in temperate pine forests. *Agricultural and Forest Meteorology* **150**, 1090–1101 (2010).

128. King, G., Fonti, P., Nievergelt, D., Büntgen, U. & Frank, D. Climatic drivers of hourly to yearly tree radius variations along a 6 °C natural warming gradient. *Agricultural and Forest Meteorology* **168**, 36–46 (2013).

129. Vogel, T., Dohnal, M., Dusek, J., Votrubova, J. & Tesar, M. Macroscopic Modeling of Plant Water Uptake in a Forest Stand Involving Root-Mediated Soil Water Redistribution. *Vadose Zone Journal* **12**, vzj2012.0154 (2013).

130. Lüttschwager, D. & Remus, R. Radial distribution of sap flux density in trunks of a mature beech stand. *Ann. For. Sci.* **64**, 431–438 (2007).

131. Aguadé, D., Poyatos, R., Rosas, T. & Martínez-Vilalta, J. Comparative Drought Responses of Quercus ilex L. and Pinus sylvestris L. in a Montane Forest Undergoing a Vegetation Shift. *Forests* **6**, 2505–2529 (2015).

132. Isarangkool Na Ayutthaya, S. *et al.* Water loss regulation in mature Hevea brasiliensis: effects of intermittent drought in the rainy season and hydraulic regulation. *Tree Physiology* **31**, 751–762 (2011).

133. Yi, K., Dragoni, D., Phillips, R. P., Roman, D. T. & Novick, K. A. Dynamics of stem water uptake among isohydric and anisohydric species experiencing a severe drought. *Tree Physiology* **37**, 1379–1392 (2017).

134. Pangle, R. E. *et al.* Methodology and performance of a rainfall manipulation experiment in a piñon–juniper woodland. *Ecosphere* **3**, art28 (2012).

135. Wilson, K. B., Hanson, P. J., Mulholland, P. J., Baldocchi, D. D. & Wullschleger, S. D. A comparison of methods for determining forest evapotranspiration and its components: sap-flow, soil water budget, eddy covariance and catchment water balance. *Agricultural and Forest Meteorology* **106**, 153–168 (2001).

136. Tajchman, S. J., Fu, H. & Kochenderfer, J. N. Water and energy balance of a forested Appalachian watershed. *Agricultural and Forest Meteorology* **84**, 61–68 (1997).
